# A review of current practice in the design and assessment of internal pilots in UK NIHR clinical trials

**DOI:** 10.1186/s13063-019-3669-9

**Published:** 2019-09-18

**Authors:** Anna Rosala-Hallas, Carrol Gamble, Jane Blazeby, Paula R. Williamson

**Affiliations:** 1 Clinical Trials Research Centre, University of Liverpool, a member of Liverpool Health Partners, Liverpool, UK; 2 Medical Research Council ConDuCT II Hub for Trials Methodology Research, NIHR Bristol Biomedical Research Centres, Population Health Sciences, Bristol Medical School, University of Bristol, Bristol, UK; 3 Medical Research Council North West Hub for Trials Methodology Research/Clinical Trials Research Centre, University of Liverpool, a member of Liverpool Health Partners, Liverpool, UK

**Keywords:** Internal pilot, Clinical trials, Methodological research

## Abstract

**Background:**

Internal pilots provide useful information which can help to optimise the running of the main trial. Although some recommendations exist in the literature for the design of internal pilots, little is known about current practice in terms of the specification and also the assessment of progression criteria. The aim of the review is to provide an overview of current practice.

**Methods:**

A cohort of clinical trials with an internal pilot, funded by the National Institute for Health Research (NIHR), Health Technology Assessment programme (HTA), extracted in 2017 was reviewed. Data were extracted from: project descriptions; summary of changes from the first stage; feedback about the full application; monitoring notes; progress report history and protocols, for information about the design and assessment of internal pilots.

**Results:**

Fifty-seven studies were reviewed. An internal pilot was first proposed in the early stages of the trial in the majority of cases. Target number for recruitment, rate of randomisation, retention/primary outcome ascertainment rate, rate of treatment adherence and consent rate were included as progression criteria. All but one study was permitted to continue to the main trial; however, 25% did not strictly meet the progression criteria. Changes were made to the design of the main trial for 25% of studies, mainly in terms of conduct of recruitment.

**Conclusions:**

This review provides insight into the process of designing and assessing internal pilots. Progression criteria are sometimes not met; however, committees involved in the reviewing process will recommend continuation to the main trial, usually accompanied by a second review or close monitoring. Recommendations are made to optimise the process.

**Electronic supplementary material:**

The online version of this article (10.1186/s13063-019-3669-9) contains supplementary material, which is available to authorized users.

## Background

Running a clinical trial can be expensive and complicated. Anticipation of hurdles at the design stage will optimise trial conduct. One method is to run a pilot trial, mirroring the larger-scale trial but with fewer participants and fewer resources. Pilot trials can impart unique insight into the day-to-day issues in running a trial and can provide a justification for progressing to, or abandoning, the full-scale trial. Different types of pilot trial exist [1–3]. An external pilot (also referred to as an external feasibility study) stands alone from the main trial and can be used to test the feasibility of elements within the trial including recruitment. Data from the external pilot are not used in the main trial analysis. An internal pilot is a phase within the main trial where data from the experience over a pre-specified period are used to determine the design of the rest of the trial, amended or not from the original proposal. Data from the internal pilot are used within the final analysis. The internal pilot should have clear pre-specified progression criteria for moving on to the main trial.

To optimise the benefit of including an internal pilot the progression criteria must be chosen carefully. Recommendations are made in the literature for developing these criteria including: basing recruitment targets on rates per centre per unit time, as opposed to an overall target number at a set time point, to account for unpredictability in site openings; inclusion of intervention adherence and cross-over; event rates; and review of missing data in terms of primary and secondary outcomes. Inclusion of both the funders and Trial Steering Committee (TSC) in the internal pilot review process is recommended [4].

In terms of choosing the pilot sites, it is advised that these must be representative of the sites to be included in the main trial [4, 5]. The protocol should detail the timing of the internal pilot analysis and outline the progression criteria [2, 4, 5].

Little is known, however, about internal pilot trials in practice [4] and few empirical studies are undertaken yet they are widely recommended by funding bodies. The aim of this study was to provide an insight into current practice regarding internal pilots in clinical trials, specifically the design and review process, by assessing a cohort of studies funded by the National Institute for Health Research (NIHR) Health Technology Assessment (HTA) programme.

## Methods

The cohort of clinical trials involving an internal pilot, funded by the NIHR HTA programme, was extracted in March 2017. Trials were identified and data extracted via a search of the NIHR Management Information System by the NIHR Evaluation, Trials and Studies Coordinating Centre (NETSCC). All trials from May 2012 that were funded and active or in the publication process with the NIHR Journals Library were included to the point of date of extraction.

After approval from the respective trial chief investigators (CIs) was obtained by NETSCC, the following information was collated: project descriptions; a summary of changes from the first stage; feedback about the full application; monitoring notes and progress report history. Protocols were accessed from the online NIHR Journals Library [6]. Development of a classification for continuation to full trial was carried out by the team and confirmation and checks were discussed with the NETSCC Research on Research (RoR) team. The classification consisted of the following categories: criteria met, pilot passed; criteria not met, pilot passed; unknown whether criteria met, pilot passed; pilot did not pass.

Data were extracted on: choice of progression criteria; the review process; the decision on continuation and any changes made to the design based on the pilot.

Data extracted were quality checked via double-data extraction, carried out for three trials (5%). The second reviewer was chosen on the basis of having had some involvement in the trial, i.e. CI or statistician, in order to assess whether the information available depicted the trial well.

Descriptive statistics were calculated using SAS Version 9.4.

## Results

Seventy-four trials were identified as eligible. Seventeen were removed from the cohort (four CIs declined, four organisation contracts offices did not reply, two CIs had not been able to consult their TSC for approval and seven did not respond to the email); a total of 57 trials comprise the cohort. For the 17 trials excluded, four had their pilots extended and the remaining 13 had passed.

The available information for each trial is summarised in Table 1. Sixteen trials had missing project descriptions, summaries of changes from the first stage and feedback about the full application, due to the trials pre-dating the development of the NETSCC Management Information System (April 2012).

**Table 1:** Details of the design of the internal pilots

	Frequency (%)	
Data sources		
Commissioning briefs	28^a^ (100%)	
Project descriptions	41 (72%)	
Summary of changes from first stage/feedback about full application	41 (72%)	
Monitoring notes	56 (98%)	
Progress report history	56 (98%)	
Protocols (current version available online)	57 (100%)	
Protocols (any previous versions available online)	37 (65%)	
Where was the internal pilot first proposed?		
	Funder-led	Researcher-led
Commissioning brief	8 (29%)	N/A
Outline	6 (27%)	14 (64%)
Outline feedback	6^b^ (27%)	7^c^ (32%)
Full application	0 (0%)	0 (0%)
Full application feedback	2 (9%)	1 (5%)
Total	22^d^ (100%)	22^d^ (100%)
Trials with progression criteria specified in the latest available version of the protocol		
Yes	36 (63%)	
No	21 (37%)	
* If yes, specified in previous available versions?*		
*Yes*	*19 (86%)*	
*No*	*3 (14%)*	
* Previous versions not available*	*14*	
Component(s) of progression criteria^e^		
Target number for recruitment within a specified period	54 (96%)	
In a specified number of sites?		
*Yes*	*4 (7%)*	
*No*	*50 (93%)*	
Retention/primary outcome ascertainment rate	16 (29%)	
Rate of treatment adherence	13 (23%)	
Rate of randomisation over time	11 (20%)	
* Rate of randomisation by site?*		
*Yes*	*8 (73%)*	
*No*	*3 (27%)*	
Number of sites recruiting	10 (18%)	
Consent rate	8 (14%)	
Number of sites opened	5 (9%)	
Proportion of eligible participants	3 (5%)	
Other^f^	4 (7%)	

^a^Denominator 28 as only applicable for the 28 funder-led trials

^b^The outline feedback suggested amendments to an additional 6 internal pilots, 4 of which were initially proposed in the commissioning brief and 2 which were proposed in the outline

^c^The outline feedback suggested amendments to an additional 8 internal pilots, all of which were initially proposed in the outline

^d^5 unknown for funder-led trials; 8 unknown for researcher-led trials; commissioning brief denominator = 28

^e^Criteria was not specified for 1 internal pilot so denominator taken as 56

^f^Number of UK participants; number receiving rescue treatments; outcome assessments conducted according to protocol; number of sites that completed the intervention course

The majority of internal pilots were first proposed in the initial stages (Table 1).

Thirty-six trials (63%) specified the progression criteria in the latest available version of the protocol and of the 22 with available previous versions, 19 (86%) specified the progression criteria in all available previous versions of the protocol.

Fifty-four trials (96%) included the target number for recruitment in their progression criteria, 16 (29%) the retention/primary outcome ascertainment rate, 13 (23%) the rate of treatment adherence, 11 (20%) the rate of randomisation, 10 (18%) the number of sites actively recruiting, 8 (14%) the consent rate, 5 (9%) the number of sites opened, 3 (5%) the proportion of eligible participants. One trial did not specify the criteria.

In the majority of cases (91%) the internal pilot followed the design agreed with the funder. Five (9%) pilots did not follow their original design: two internal pilots were extended due to poor recruitment, one of which also amended the progression criteria to remove the first criterion of the number of centres recruiting their first participant and revised the criterion of a target number to be in terms of average patients recruited per site per month; three were delayed – one due to recruitment being put on hold, one due to a delay in recruiting the trial manager and one due to early delays with no further information given (Table 2).

**Table 2:** Details of the assessment of the internal pilots

	Frequency (%)
Design followed? (Yes/No)^a^	
Yes	49 (91%)
No	5 (9%)
*Extended due to poor recruitment*	*2 (40%)*
*Internal pilot delayed*	*3 (60%)*
Committees involved in review^b^	
IDSMC only	3 (6%)
TSC only	17 (35%)
IDSMC, TSC	23 (47%)
IDSMC, TSC, TMG	2 (4%)
TSC, sponsor	3 (6%)
Continuation to main trial? (Yes/No)	
Yes, met criteria	33 (58%)
Yes, did not meet criteria	14 (25%)
*Underwent a second review*^*c*^	*5 (36%)*
*Continued with close monitoring*	*3 (21%)*
*Recovery plan put in place*	*3 (21%)*
*No reported actions taken*	*4 (29%)*
Yes, unclear whether criteria met	9 (16%)
Decision on hold	1 (2%)
No	0 (0%)
Number of planned pilot sites given?^d^ (Yes/No)	
Yes	44 (79%)
No	12 (21%)
* If yes, specifically named?*	
*Yes*	*16 (36%)*
*No*	*28 (64%)*
Number of planned pilot sites	
Median	6 (5, 10)
(interquartile range (IQR))	
Min, Max	2, 80
Number of planned sites for full trial^f^	
Median (IQR)	20 (13, 30)
Min Max	3, 300
Adherence to plan regarding number of pilot sites?^e^ (Yes/No)	
Yes	28 (72%)
No	10 (26%)
*Opened further sites*	*6 (60%)*
*Opened fewer sites*	*4 (40%)*
Missing	6

^a^Design not given in the protocol/proposal for 1 trial; unclear from the available information for 2 trials

^b^Not given for 8 trials

^c^Second review involved revised recruitment targets; 1 trial underwent close monitoring alongside second review

^d^Not applicable for 1 trial – no sites since recruitment takes place online

^e^Number of planned pilot sites not given for 12 trials

^f^Including pilot sites; not given for 2 trials

*IDSMC* Independent Data and Safety Monitoring Committee, *TSC* Trials Steering Committee, *TMG* Trial Management Group

Alongside the funder, the TSC was most commonly reported to be involved in the reviewing of the progression criteria, reviewing 94% of trials. No discussion of, or rationale for which committees were chosen to review the results, or in what order, was given for any study.

Fifty-six trials (98%) continued to the full trial, although 14 did not meet the pre-specified progression criteria (see Additional file 1: Table S1); 5 (36%) of which underwent a second review prior to continuation where new criteria were agreed by the trialists and the funder, 3 (21%) put a recovery plan in place and 3 (21%) continued with close monitoring. For nine trials it was unclear whether the criteria were met. For one trial the decision on continuation was on hold at the time of data extraction due to delays in the pilot period.

Forty-four trials (79%) gave a planned number of sites to participate in the internal pilot, 16 (36%) of which specifically named the sites in the protocol.

Twenty-eight (74%) trials adhered to the plan regarding the number of sites. Ten (26%) trials did not adhere to the plan: six opened more sites due to poor recruitment and four opened fewer sites than planned. There was no information on adherence to pilot site choice for six of the trials.

Changes were made to the design of the main trial as a result of the internal pilot in 14 (25%) trials; see Table 3.

**Table 3:** Details of the outcomes of internal pilots

Changes made to the design of the main trial? (Yes/No)	Frequency (%)
Yes, conduct of recruitment	10 (18%)
*Inclusion criteria widened*	*2 (20%)*
*Increased number of sites*	*2 (20%)*
*The predicted recruitment rate for the rest of the trial was amended*	*1 (10%)*
*Recruitment extended for the main trial*	*1 (10%)*
*Reduced the number of arms in the trial and the sample size*	*1 (10%)*
*TSC recommended sample size re-estimation given observed complication rate difference*	*1 (10%)*
*Simplified of inclusion/exclusion criteria; shortened of case report forms*	*1 (10%)*
*Widened inclusion criteria; increased number of sites*	*1 (10%)*
Yes, impact on outcome data	1 (2%)
*Clarifications made to the protocol; flexibility added to location of outcome assessments*	*1 (100%)*
Yes, in terms of both recruitment and outcome data	1 (2%)
*Change of eligibility criteria; change to secondary outcomes; inclusion of a sub-trial*	*1 (100%)*
Yes, no further information given	2 (4%)
No	43 (75%)
Total	57 (100%)

*TSC* Trial Steering Committee

## Discussion

There is increasing use of an internal pilot design to optimise main trial conduct. This review examined the design of internal pilots and how progression criteria were chosen and used to inform progression to the main study. Of 57 trials from an HTA cohort the majority were first proposed in the early stages whether of funder- or researcher-led trials. Although most trials specify the progression criteria in the protocol, a considerable proportion of trials do not (37%), despite recommendations in the literature. It was clear from the included trials that progress to the main trial includes a number of steps and can at times be complex. The decision-making process from the internal pilot to the main trial included a multidisciplinary approach, whereby the funders, trialists, TSC, Independent Data and Safety Monitoring Committee (IDSMC), Trial Management Group (TMG) and sponsor contributed to the progression of a trial. If the reporting of the progression criteria were more transparent in the protocol, this could help to eliminate any uncertainties during the internal pilot stage.

The pilot and feasibility trials extension to the Consolidated Standards of Reporting Trials (CONSORT) 2010 Statement states that objectives of the pilot study should be stipulated in the trial report [7]. The objectives were clear for the trials in this review but none provided a specific rationale for choosing the progression criteria; giving the rationale within the protocol may aid decision-making if certain criteria are not met.

Previous work has examined how feasibility trials (i.e. standalone randomised pilot studies) or ‘external pilots’ funded by the NIHR Research for Patient Benefit (RfPB) programme progressed to main studies. In a cohort of 89 trials which had closed by May 2016, 20 (30%) were judged ‘not feasible’, of which 6 (7%) were intending to apply for funding for the main trial [8]. However, in the current review all but one internal pilot, which was still under review at the time of analysis, continued to the main trial, even when progression criteria had not been met. This is reassuring as a standalone feasibility study is deliberately performed when the level of success for the main trial is much more uncertain than when an internal pilot is undertaken. However, our findings also suggest that a nuanced approach is used to assess the viability of trial when reviewing an internal pilot which can include a second review, ongoing monitoring and changes to the design of the main trial in terms of recruitment and outcomes. Although some of the trials underwent further reviews, none specified this in the protocol; consideration should be given to include plans for this in the protocol when pre-specifying the progression criteria in the event that criteria are not met. Sometimes changes to the trial design are invoked by the results of the internal pilot; in such cases these changes should be documented clearly within the trial report [6].

The majority (77%) of CIs agreed to share their monitoring data and only 4 (5%) CIs explicitly declined; this is important for a move towards more transparent reporting of the decision-making process. Given that the pilots in excluded trials either passed the progression criteria or had their pilot phases extended there was no bias in terms of non-response due to non-progression to the main trial.

One limitation of the review is that the data extraction came from various, sometimes incomplete, sources. An extension of the work would be to contact the respective CIs to ask for a review of the data extracted for their trial to confirm validity or to add where data may have been missing. This was not carried out for this review as firstly, it would have been too time-consuming and, secondly, it was assumed that the information provided was sufficient to give an overall summary which was confirmed by the double-data extraction. These findings may not necessarily be extrapolated to other funding bodies; thus, this work could be extended to include trials from other NIHR programmes as well as other funding bodies to compare progression criteria and progress across funders.

Seventeen trials had a qualitative element which either ran alongside the internal pilot or was incorporated within it. Objectives of the qualitative study were mainly focussed around understanding the barriers and facilitators to recruitment and the consent process, including patients’ motives for giving or refusing consent. There were qualitative elements in some of the other included trials; however, these were either scheduled to take place pre or post internal pilot. Further work can be done to assess the impact of these qualitative elements on the decision to proceed to the main trial.

## Conclusions

This review has provided an overview of current practice in planning and carrying out internal pilots and gives a behind-the-scenes insight into the review process with the hopes of moving towards transparent reporting in the design and analysis of internal pilots. Protocols should state progression criteria and give consideration to reporting the rationale of each criterion. Consideration should also be given to add plans for second reviews of the progression criteria to the protocol and any changes made to the trial design as a result of the internal pilot.

## Additional file

Additional file 1: **Table S1.** Further details on progression criteria not met. **Table S2.** List of included trials. (DOCX 27 kb)

## Data Availability

The datasets generated and/or analysed during the current study are available from the corresponding author on reasonable request.

## Abbreviations

CI: Chief investigator
HTA: Health Technology Assessment
IDSMC: Independent Data and Safety Monitoring Committee
NETSCC: NIHR Evaluation, Trials and Studies Coordinating Centre
NIHR: National Institute for Health Research
RfPB: Research for Patient Benefit
RoR: Research on Research
TMG: Trial Management Group
TSC: Trial Steering Committee
