# Supplementary material for: A review of current practice in the design and assessment of internal pilots in UK NIHR clinical trials
Source: Trials. 2019 Sep 18;20:571. doi: 10.1186/s13063-019-3669-9 (PMC6751663; doi:10.1186/s13063-019-3669-9)
Supplement: Supplementary file 1 — Table S1. Further details on progression criteria not met. Table S2. List of included trials. (DOCX 27 kb) [file 13063_2019_3669_MOESM1_ESM.docx]

Additional file 1

Table S1. Further details on progression criteria not met

| **Further details on progression criteria not met** | **N (%)** |
| --- | --- |
| Recruitment rate was 50% of target. No further information given for remaining two targets. | 1 (14%) |
| 40% of target number of sites opened. Other progression criteria were not assessed due to being based on having all sites open. Participant recruitment target element had been met in one centre and so approval for continuation was granted. | 1 (14%) |
| Recruited 38% of target. | 1 (14%) |
| Recruited 67% of target. | 1 (14%) |
| 45% of target site months achieved.  Recruitment rate target was met.  Recruited 67% of target. | 1 (14%) |
| The results were “neither a clear success nor failure”; no further information. | 1 (14%) |
| Recruited 30% of target.  24% of target site months achieved.  43% of target number of sites opened. | 1 (14%) |
| Criteria not met; no further information | 7 (50%) |
| **Total** | **14 (100%)** |

Table S2. List of included trials

| **NETSCCID** | **Title** | **Chief Investigator** |
| --- | --- | --- |
| 12/10/18 | A randomised controlled trial of the ketogenic diet in the treatment of epilepsy in children under the age of two years | Cross, Judith |
| 13/95/10 | Probiotic to Reduce Infections iN CarE home reSidentS (PRINCESS) | Butler, Christopher |
| 09/104/19 | A controlled study of the effectiveness of breathing training exercises taught by a physiotherapist by either instructional videos/DVDs/internet download or by face-to-face sessions in the management of asthma in adults | Thomas, Mike |
| 09/127/53 | Gastric Bypass, adjustable gastric Banding or Sleeve gastrectomy surgery to treat severe and complex obesity: a multi-centre randomised controlled trial (The By-Band-Sleeve Study) | Blazeby, Jane |
| 09/165/01 | Self-Management education for adults with poorly controlled epILEpsy (SMILE) A Randomised Controlled Trial | Ridsdale, Leone |
| 10/50/49 | Palliative radiotherapy in addition to self-expanding metal stent for improving outcomes of dysphagia and survival in advanced oesophageal cancer: ROCS (Radiotherapy after Oesophageal Cancer Stenting) Study | Adamson, Douglas |
| 10/57/14 | A multi-centre randomised placebo-controlled trial of prophylactic enteral supplementation with bovine lactoferrin to prevent late-onset invasive infection in very preterm or very low birth weight infants. | McGuire, William |
| 10/57/20 | A randomised controlled trial of standard-of-care wound management versus negative pressure wound therapy in the treatment of adult patients with an open fracture of the lower limb | Costa, Matthew |
| 10/137/01 | Therapeutic Interventions for Stones of the Ureter (TISU): a multicentre randomised controlled trial of extracorporeal shockwave lithotripsy, as first treatment option, compared with direct progression to ureteroscopic retrieval, for ureteric stones. | McClinton, Samuel |
| 11/01/04 | HALT-IT trial (Haemorrhage Alleviation with Tranexamic acid–Intestinal System), a large randomised placebo controlled trial among patients with acute gastrointestinal haemorrhage of the effects of tranexamic acid on death and transfusion requirement | Roberts, Ian |
| 11/26/05 | RATULS: Robot Assisted Training for the Upper Limb after Stroke | Rodgers, Helen |
| 11/36/16 | The Low Risk DCIS (ductal carcinoma in situ) Trial | Wallis, Matthew |
| 11/36/41 | Allopurinol and cardiovascular outcomes in patients with ischaemic heart disease | Mackenzie, Isla |
| 11/58/15 | A randomised, double-blind placebo controlled trial of the effectiveness of low dose oral theophylline as an adjunct to inhaled corticosteroids in preventing exacerbations of chronic obstructive pulmonary disease. | Price, David |
| 11/65/01 | Randomised controlled trial of silk therapeutic clothing for the long-term management of eczema in children (CLOTHES Trial: CLOTHing for the relief of Eczema Symptoms) | Thomas, Kim |
| 11/67/01 | Cancer And Venous Access (CAVA) – a randomised controlled trial with associated qualitative research of long term venous access devices for the delivery of chemotherapy: Implantable venous access ports versus tunnelled central lines versus peripheral inserted central catheters. | Moss, Jon |
| 11/72/01 | Antibiotic treatment for intermittent bladder catheterisation: A randomised controlled trial of once daily prophylaxis (The AnTIC study) | Pickard, Robert |
| 11/92/15 | Outcome after Selective Early Closure of Ductus Arteriosus in Extremely Preterm Babies (Baby-OSCAR trial) | Gupta, Samir |
| 11/94/01 | A cluster randomised controlled trial of a behavioural change package to prevent hand dermatitis in nurses working in the National Health Service | Madan, Ira |
| 11/106/01 | Male synthetic sling versus Artificial urinary Sphincter Trial for men with urodynamic stress incontinence after prostate surgery: Evaluation by Randomised controlled trial (MASTER) | Abrams, Paul |
| 11/114/01 | PRE-EMPT: Preventing Recurrence of Endometriosis by Means of long acting Progestogen Therapy | Bhattacharya, Siladitya |
| 11/129/109 | Tranexamic acid for hyperacute primary Intracerebral Haemorrhage (TICH-2) | Sprigg, Nikola |
| 11/136/04 | For patients with a displaced fracture of the distal tibia, is there a clinical and cost-effectiveness difference between ‘locking’ plate fixation and intramedullary nail fixation? (FixDT) | Costa, Matthew |
| 11/153/01 | BATHE (Bath Emollients for Treatment of cHildhood Eczema) | Santer, Miriam |
| 12/26/01 | Cognitive behavioural therapy vs standardised medical care for adults with Dissociative non-Epileptic Seizures: A multi-centre randomised controlled trial (CODES) | Goldstein, Laura |
| 12/28/05 | STEPWISE: STructured lifestyle Education for People WIth Schizophrenia | Holt, Richard |
| 12/29/01 | A pragmatic adaptive sequential placebo controlled randomised trial to determine the effectiveness of Glycerin trinitrate for retained placenta (Got-it trial) | Denison, Fiona |
| 12/33/12 | Primary care use of a C-Reactive Protein (CRP) Point of Care Test (POCT) to help target antibiotic prescribing to patients with Acute Exacerbations of Chronic Obstructive Pulmonary Disease (AECOPD) who are most likely to benefit (The PACE Study) | Butler, Christopher |
| 12/35/38 | Treatment of Advanced Glaucoma Study (TAGS): A multicentre randomised controlled trial comparing primary medical treatment with primary trabeculectomy for people with newly diagnosed advanced glaucoma | King, Anthony |
| 12/35/45 | Multicentre randomised controlled trial to compare the clinical and cost-effectiveness of a ‘vein bypass first’ with an ‘endovascular first’ revascularisation strategy for severe limb ischaemia due to infrageniculate arterial disease (Bypass v Angioplasty in Severe Ischaemia of the Leg, BASIL-2) | Bradbury, Andrew |
| 12/35/57 | Randomised Evaluation of Surgery with Craniectomy for patients Undergoing Evacuation of Acute Subdural Haematoma (RESCUE-ASDH) | Hutchinson, Peter |
| 12/35/64 | A randomised controlled trial of adjunctive intraocular and periocular steroid (triamcinolone acetonide) versus standard treatment in eyes undergoing vitreoretinal surgery for open globe trauma | Charteris, David |
| 12/127/134 | Emergency Treatment with Levetiracetam or Phenytoin in Status Epilepticus in Children – an open label randomised controlled trial | Appleton, Richard |
| 12/140/01 | Urodynamics for Prostate Surgery Trial; Randomised Evaluation of Assessment Methods (UPSTREAM) for diagnosis and management of bladder outlet obstruction in men | Drake, Marcus |
| 12/142/07 | Early detection of neovascular age-related macular degeneration | Chakravarthy, Usha |
| 12/167/02 | PREVenting infection using Antibiotic Impregnated Long lines (PREVAIL) | Gilbert, Ruth |
| 12/167/135 | A randomised controlled trial to examine the efficacy of e-cigarettes compared with nicotine replacement therapy, when used within the UK stop smoking service | Hajek, Peter |
| 12/190/05 | Cognitive Rehabilitation for Attention and Memory in people with Multiple Sclerosis (CRAMMS) | Lincoln, Nadina |
| 12/196/08 | COmmunity based Rehabilitation after Knee Arthroplasty (CORKA) | Barker, Karen |
| 12/201/09 | Stratified Care for Patients with Sciatica and Suspected Sciatica in Primary Care: A randomised trial (the SCOPiC trial - SCiatica Outcomes in Primary Care) | Foster, Nadine |
| 12/201/10 | Multi-centre randomised control trial comparing the clinical and cost effectiveness of trans-foraminal epidural steroid injection to surgical microdiscectomy for the treatment of chronic radicular pain secondary to prolapsed intervertebral disc herniation(NErve Rootblock VErsus Surgery:NERVES) | Wilby, Martin |
| 13/04/03 | VIdeo assisted thoracoscopic lobectomy versus conventional Open LobEcTomy for lung cancer, a multi-centre randomised controlled trial with an internal pilot. The VIOLET Study | Lim, Eric |
| 13/04/22 | An open randomised trial of the Arabin pessary to prevent preterm birth in twin pregnancy - STOPPIT - 2 | Norman, Jane |
| 13/04/107 | Cerclage Suture Type for an Insufficient Cervix and its effect on Health outcomes (C-STICH) | Toozs-Hobson, Philip |
| 13/04/108 | The RAPID-CTCA trial (Rapid Assessment of Potential Ischaemic Heart Disease with CTCA) The role of early CT Coronary Angiography in the evaluation, intervention and outcome of patients presenting to the Emergency Department with suspected or confirmed Acute Coronary Syndrome | Gray, Alasdair |
| 13/15/02 | A randomised, double blind, placebo-controlled trial of a two-week course of dexamethasone for adult patients with a symptomatic Chronic Subdural Haematoma (Dex-CSDH trial) | Hutchinson, Peter |
| 13/26/01 | Multi-centre randomised controlled trial with economic evaluation and nested qualitative study comparing early structured physiotherapy versus manipulation under anaesthesia versus arthroscopic capsular release for patients referred to secondary care with a frozen shoulder (Adhesive Capsulitis) | Rangan, Amar |
| 13/84/10 | Exercise to prevent shoulder conditions in patients undergoing breast cancer treatment. The PRevention Of Shoulder Problems Study (PROSPER) | Bruce, Julie |
| 13/88/10 | Electronically-delivered, multi-component interventions to reduce unnecessary antibiotic prescribing in primary care. A cluster randomised trial using electronic health records (eCRT2) | Gulliford, Martin |
| 13/115/62 | UK Study of tendo Achilles Rehabilitation – multicentre randomised clinical trial (UK STAR) | Costa, Matthew |
| 13/115/82 | E-FREEZE: a randomised controlled trial evaluating the clinical and cost-effectiveness of a policy of freezing all embryos followed by thawed frozen embryo transfer, compared with a policy of fresh embryo transfer in women undergoing in-vitro fertilization | Maheshwari, Abha |
| 13/143/02 | pRotective vEntilation with veno-venouS lung assisT in respiratory failure.  The REST Trial | McAuley, Danny |
| 13/153/04 | Liver Resection Surgery Versus Thermal Ablation for Colorectal LiVer MetAstases | Davidson, Brian |
| 14/08/45 | A Multicentre Randomised Controlled Trial of Induced Endometrial Trauma In Women Undergoing First Time In Vitro Fertilisation (IVF) | Metwally, Mostafa |
| 14/49/34 | An online randomised controlled trial to evaluate the clinical and cost effectiveness of a peer supported self-management intervention for relatives of people with psychosis or bipolar disorder: Relatives Education And Coping Toolkit (REACT) | Lobban, Fiona |
| 14/186/01 | Mentalization for Offending Adult Males (MOAM) | Fonagy, Peter |
| 14/192/71 | A randomised controlled trial comparing the clinical effectiveness and cost-effectiveness of laparoscopic cholecystectomy compared with observation/conservative management for preventing recurrent symptoms and complications in adults with uncomplicated symptomatic gallstones (C-Gall) | Ahmed, Irfan |
| 14/199/14 | WHIST - Wound Healing In Surgery for Trauma | Costa, Matthew |
